# Trust and trade: patient perspectives on the ethics of real-world data monetisation

**DOI:** 10.3389/frhs.2026.1771744

**Published:** 2026-04-17

**Authors:** Alexandros Sagkriotis

**Affiliations:** Independent Consultant in Real-World Evidence and Health Data Science, Helios Academy Limited | Where Science Meets Compassion, London, United Kingdom

**Keywords:** consent, data monetisation, ethics, evidence integrity, governance, patient trust, real-world data (RWD), real-world evidence (RWE)

## Abstract

Real-world data (RWD) and real-world evidence (RWE) are increasingly used to inform regulatory decisions, health technology assessment, and health system planning. However, patients whose data underpin these activities often experience limited transparency or benefit when their information is monetised. While regulatory and HTA frameworks emphasise methodological rigor and analytical transparency, they provide limited guidance on fairness, reciprocity, and legitimacy from a patient perspective. This Policy Brief examines this governance gap and argues that evidence integrity must extend beyond technical standards to include ethical stewardship and public trust. Drawing on policy contexts from UK, EU, and North America, it proposes five pragmatic safeguards to strengthen transparency, accountability, and patient-centred governance in secondary data use, supporting the sustainability and legitimacy of RWE infrastructures as data initiatives expand.

## Introduction

Real-world data (RWD) are firmly embedded in regulatory science and health policy, with the European Medicines Agency (EMA) and the U.S. Food and Drug Administration (FDA) formally recognising real-world evidence (RWE) as a key element across the product lifecycle (1, 2). The Data Analysis and Real World Interrogation Network in the European Union (DARWIN EU) exemplifies the move from principles to execution by routinely generating regulator-led studies across Europe (3). Yet these frameworks prioritise methodological adequacy and transparency; they do not meaningfully adjudicate the fairness of monetised secondary use from a patient's point of view.

**Table 1:** Scope of current RWE and HTA frameworks vs. ethical dimensions.

Framework	Methodological rigor	Transparency	Monetisation governance	Patient benefit/reciprocity
EMA RWE Framework	Yes	Yes	No	No
FDA RWE Framework	Yes	Yes	No	No
HTA Tools (e.g., REQueST)	Yes	Yes	No	No

This commentary recognises the scientific value and necessity of real-world evidence generation and focuses on a critical governance blind spot: how ethical legitimacy, patient trust, and reciprocity are addressed—or overlooked—when health data are transformed into economic value.

Concurrently, the European Health Data Space (EHDS) introduces a legal infrastructure for the primary and secondary use of electronic health data (in force from March 26, 2025), explicitly enabling reuse for research and policy while strengthening individual control (4). This represents a pivotal opportunity to align governance with public expectations around trust and benefit-sharing.

This paper builds on established methodological standards for RWE and extends the discussion by addressing a more fundamental set of questions:

- How do current regulatory and health technology assessment (HTA) frameworks address—or fail to address—the fairness of secondary use and monetisation of RWD?
- To what extent are patients informed about, and comfortable with, their data being transformed into economic value for institutions, companies, or individual careers?
- What safeguards could strengthen trust, reciprocity, and legitimacy in the secondary use of health data?

The objective is to extend the concept of evidence integrity beyond methodological rigor. These research questions are addressed through a structured qualitative synthesis of policy frameworks, patient perception studies, and illustrative governance cases, rather than through primary empirical analysis.

From a theoretical standpoint, this paper draws on *relational ethics*—which emphasises reciprocity and interdependence between data contributors and data users—and on the *social responsibility of science*, which situates evidence generation within collective obligations toward public good and democratic participation. To delineate the boundaries of this commentary, the analysis concentrates on ethical and relational dimensions of secondary data monetisation from a patient perspective. The analysis focuses specifically on ethical and relational dimensions of secondary data monetisation from a patient perspective, while recognising that related themes—such as clinical urgency, operational efficiency, and broader societal objectives—remain important complementary considerations beyond the scope of this work. These frameworks guide the interpretation of trust, fairness, and legitimacy throughout the paper.

## Conceptual framework and operational definitions

Trust, reciprocity, legitimacy, and evidence integrity are central constructs in this commentary but are often used inconsistently across the literature. For clarity, they are defined here as analytical lenses guiding interpretation rather than as quantitatively measured variables.

**Trust** is understood as the expectation that institutions will handle patient data responsibly, transparently, and in alignment with patient interests.

**Reciprocity** refers to the expectation that value generated from patient data is returned in a meaningful way to patients or society, typically at a collective rather than individual level.

**Legitimacy** denotes the perceived fairness and acceptability of data use, particularly when data are used for secondary or commercial purposes.

**Evidence integrity**, as used in this commentary, extends beyond methodological rigor to include ethical stewardship, transparency, and fairness in data governance.

In this analysis, these constructs are not operationalised through formal measurement but are applied qualitatively to interpret patterns across patient surveys, policy frameworks, and governance case studies.

## Approach and source selection

This article is a structured narrative policy commentary rather than a systematic review. It synthesises evidence from multiple source types to examine ethical and governance dimensions of real-world data monetisation.

Sources were purposively selected based on their relevance to patient trust, governance, and data use, including:

- published patient surveys and systematic reviews on public attitudes to data use,
- regulatory and policy frameworks (e.g., EMA, FDA, EHDS),
- peer-reviewed literature on data governance and ethics,
- and well-documented case studies illustrating governance challenges.

The selection approach was **deliberately purposive and policy-oriented rather than exhaustive**, aiming to capture recurring and policy-relevant themes rather than provide a comprehensive literature review.

A structured narrative synthesis approach was applied, prioritising policy relevance and thematic consistency across sources. As such, the analysis is intended to provide an informed and policy-oriented interpretation of the evidence landscape.

## What current frameworks cover—and what they miss

Before examining governance gaps, it is important to define that the term “monetisation” in this commentary refers to economic value generation from secondary-use health data by public institutions, private companies, data intermediaries, and technology vendors.

Regulatory and HTA-related guidance has matured rapidly: STaRT-RWE standardises protocol transparency (5); HARPER provides a harmonised protocol template (6); and the EUnetHTA REQueST tool articulates registry quality criteria, while maintaining a primary focus on methodological validity and reporting, with limited explicit consideration of how economic value is distributed or communicated to patients (7). In practice, monetisation occurs through several mechanisms: (i) health systems or EHR vendors licensing de-identified datasets; (ii) private data brokers aggregating and selling longitudinal health data; (iii) pharmaceutical and med-tech companies purchasing access for research and evidence generation; and (iv) digital platforms generating value through algorithm development or proprietary analytics.

FDA's RWE framework and its 2025 program updates reiterate definitions and decision contexts for using RWD in approvals and labelling changes—again, squarely methodological. The result is a governance landscape that improves science but leaves the ethics of monetisation and reciprocity largely to institutional policies and contracts (2).

This comparison illustrates that while existing frameworks strongly address methodological and reporting standards, they do not systematically incorporate considerations of economic value flows, reciprocity, or patient-centred legitimacy.

## The patient trust gap

Survey work in the United Kingdom (UK) consistently indicates high trust in the National Health System (NHS) as a steward of data, but markedly lower trust in pharmaceutical and technology companies. In 2024, NHS Digital reported 72%–83% trust in the NHS to keep data secure; curated evidence reviews from Understanding Patient Data (2021–2024) similarly document both broad support for data use and persistent unfamiliarity with secondary uses. Public preference skews towards de-identified data and transparency about purpose (8).

Findings from a 2024 systematic review suggest that public discomfort with commercial access remains high, especially when data are used for marketing or insurance purposes. The review also highlights that willingness to share for third-party uses hinges on trust, perceived public benefit, and clear safeguards (9). Earlier UK surveys confirm these trends, with NHS stewardship trusted far more than corporate actors (8). These findings reinforce the central argument of this commentary: patients distinguish sharply between public-benefit–oriented data use and commercial or opaque models of data value extraction.

Global consumer research echoes this. Deloitte's 2024–2025 surveys find rising scepticism toward generative AI in health contexts, driven by distrust in outputs and unease about data handling—signals that any monetised data ecosystem must take seriously (10).

## Case illustrations: when opacity erodes legitimacy

While both the DeepMind–Royal Free and NHS data platform debates reveal governance fragility, the DeepMind case illustrates in detail how the absence of early engagement erodes legitimacy. Analysing this case through the lens of *relational ethics* clarifies that the failure was not merely procedural but relational—patients were treated as data sources rather than moral partners.

### DeepMind–royal free (UK)

In 2017 the UK Information Commissioner's Office concluded that Royal Free NHS Foundation Trust failed to comply with data protection law in sharing 1.6 million patient records with Google's DeepMind to develop and test the Streams app, citing inadequate patient information. The episode is now a canonical example of “legal-process first, engagement later” and its reputational cost (11).

### NHS national data platform and pricing debates

In parallel with NHS England's Federated Data Platform build-out, policy discussions have explored a national health data service and pricing structures to recover costs of access. Editorials warn that perceived private profit from NHS data could undermine trust without visible public benefit and transparency (12).

Both cases illustrate what happens when public data are treated primarily as assets rather than as contributions from individuals — people who may rightfully feel, “this is my data”, yet are rarely consulted on its use.

These cases are selected to illustrate salient governance dynamics observed in publicly documented settings, providing insight into how transparency and engagement influence legitimacy in practice.

## Monetisation: ethics and economics

Health systems, electronic health record (EHR) vendors, and third-party platforms increasingly treat de-identified data as an asset class. Recent scholarship documents data collection and commercialisation practices in primary care record industries, and ethics papers debate whether and when for-profit secondary use of publicly generated data is acceptable. Publics tend to support research-oriented reuse with clear public benefit, but react negatively to opaque commercial models (13). This commentary uses “commercialisation” and “monetisation” to denote value flows—financial or strategic—generated from patient data, regardless of whether value accrues to public bodies, private companies, or hybrid partnerships.

For analytical clarity, monetisation can be differentiated into three broad categories:

(i) **direct financial exchange**, such as licensing of datasets;
(ii) **indirect value creation**, such as development of proprietary algorithms or digital tools; and
(iii) **cost-recovery models**, where access fees are used to sustain data infrastructure rather than generate profit.

These categories reflect distinct governance, ethical, and policy considerations and should not be treated as equivalent.

In the UK, British Medical Journal (BMJ) commentary has cautioned against “selling NHS patient data” without clear benefit-sharing and transparent governance—again reflecting a legitimacy rather than a pure privacy concern (14). The National Data Guardian's 2023–24 report similarly centres “demonstrably trustworthy” use as essential to public confidence (15).

Viewed through *social responsibility ethics*, the monetisation of health data raises questions not of ownership alone but of distributive justice—who benefits, and who bears the moral cost of data extraction.

## From method to meaning: expanding “evidence integrity”

Scientific transparency tools (STaRT-RWE, HARPER, REQueST) should be complemented by trust-building practices that speak to meaning for contributors: who profits, who governs, and who benefits. Absent this, even lawful, de-identified reuse may fail the legitimacy test—especially at scale or when private actors are central (16). Further discussion of secondary use under the European Health Data Space (EHDS) and privacy-enhancing technologies is provided by van Drumpt et al. (17).

The five safeguards proposed in this commentary were identified through the convergence of three analytical inputs:

(i) recurring concerns identified in patient perception studies, particularly regarding transparency, trust, and benefit-sharing;
(ii) documented governance challenges and policy debates, including high-profile case studies such as DeepMind–Royal Free and NHS data access discussions; and
(iii) existing accountability mechanisms in adjacent domains, such as clinical trial registration, public involvement frameworks, and transparency initiatives.

These safeguards were prioritised based on their feasibility, cross-jurisdictional applicability, and alignment with existing regulatory and health system infrastructures.

## Five pragmatic safeguards

Building on the legitimacy principles outlined above, the following five safeguards operationalise *relational ethics* and the *social responsibility of science* in practice. They translate fairness, reciprocity, and accountability into concrete governance mechanisms that extend beyond compliance. These safeguards are designed to address patient-centred legitimacy concerns specifically in contexts where data generate economic value for institutions or companies; they do not aim to regulate clinical care use or direct public-health emergencies.

(1) **Plain-language, layered consent (or notification) for secondary uses.** Consent materials should explain what kinds of secondary use and monetisation exist, by whom, and with what controls, aligned to EHDS guardrails and national opt-out regimes (4).
(2) **Mandatory disclosure of monetisation models.** Public registries of data access agreements (who accessed, for what, value exchanged) would normalise transparency and enable audit—akin to trial registration for methods (18).
(3) **Governance with patient representation.** Data access boards for secondary use should include trained patient/public members with real voting rights. This is consistent with the National Data Guardian's emphasis on “demonstrably trustworthy” use (15).
(4) **Visible benefit-sharing.** Where commercial value is created from public data, a defined proportion should be reinvested into patient support, patient associations, public health, digital tools, or the data infrastructure itself. Ongoing UK work on pricing/cost-recovery shows how models can be designed to avoid perceptions of “selling” while still covering costs (12).
(5) **Protocol registration and transparency.** All non-interventional studies using patient data—whether for HTA, regulatory submissions, or scientific communication—should be registered in publicly accessible databases such as the EU PAS Register, ClinicalTrials.gov, or ENCePP (19–21). Registration of objectives, endpoints, and analysis plans creates a transparent record that reduces selective reporting, clarifies intent, and enhances accountability. This expectation should extend to both submission-grade and non-submission RWE, ensuring that the use of patient data is always visible and auditable (22, 23).

Together, these steps do not supplant methodological standards; they expand the meaning of “evidence integrity” to include fairness, reciprocity, and legitimacy—anchoring ethical trust as a measurable dimension of scientific quality.

## Legitimate secondary uses

Access by industry to patient-level data for purposes such as HTA submissions, regulatory evidence generation, and peer-reviewed scientific communication should be regarded as legitimate and essential. These activities contribute to transparency in decision-making, accelerate patient access to innovative therapies, and improve clinical practice.

However, legitimacy requires that such uses are conducted within the same framework of safeguards: plain-language consent, disclosure of access agreements, patient-inclusive governance, visible benefit-sharing, and mandatory registration of protocols in publicly accessible registries such as the EU PAS Register, ClinicalTrials.gov, or ENCePP. Without these guardrails, even necessary evidence generation risks being perceived as exploitation rather than collaboration.

These legitimate uses set the ethical boundary conditions for the five safeguards proposed below.

## Discussion

The analysis presented here highlights a structural blind spot in the governance of RWE. Regulatory and HTA frameworks have succeeded in advancing methodological rigor, transparency, and data quality, but they remain silent on fairness, reciprocity, and benefit-sharing. This commentary synthesises recurring themes from surveys, qualitative studies, and governance cases to provide a structured perspective on how trust dynamics manifest across health data ecosystems. Surveys and case studies suggest that while patients broadly support data use for public benefit, they are consistently uneasy about opaque commercial access and monetisation. This trust gap poses a risk not only to the legitimacy of individual initiatives but to the credibility of RWE as a scientific field. Consistency across findings was assessed qualitatively through convergence of observations from independent sources, including national surveys, systematic reviews, and cross-country policy analyses. While no formal meta-synthesis was conducted, repeated patterns—particularly regarding trust asymmetry and concerns about commercial data use—were identified across multiple contexts.

At its core, this blind spot reflects a tension between methodological integrity—how well evidence is generated—and relational integrity—how fairly data relationships are governed. *Relational ethics* provides a lens through which this imbalance can be addressed. It frames patients not as passive data sources but as moral agents who participate in a continuous exchange of trust, expectation, and accountability. From this perspective, transparency is not a bureaucratic checkbox but an act of recognition and respect, and consent becomes an ongoing dialogue rather than a one-time procedural formality.

The DeepMind–Royal Free case serves as a vivid example of what happens when these relational dimensions are ignored. Despite legal authorisation, the absence of patient engagement created a perception of data extraction rather than partnership. Reinterpreting such cases through *relational ethics* suggests that failures in legitimacy are rarely technical—they are relational, arising when institutions treat data ownership as property instead of stewardship. Rebuilding this moral relationship requires new norms of dialogue, reciprocity, and visible accountability.

The *social responsibility of science* extends this reasoning from the interpersonal to the societal level. Scientific and commercial actors alike benefit from a social licence granted by citizens who share data in good faith. That licence carries duties: to reinvest value into patient communities, to democratise access to insights derived from shared data, and to ensure that monetisation does not become exploitation. Reinvestment in public infrastructure, open methods, and digital literacy programmes are therefore not peripheral acts of goodwill but expressions of moral reciprocity (24, 25). The proposed safeguards operationalise these duties in practical terms.

The safeguards proposed—transparent consent, disclosure of monetisation, patient representation in governance, visible benefit-sharing, and mandatory protocol registration— represent practical, implementable steps already reflected in related domains, including trial registration and public involvement in research governance. Each safeguard reflects a dimension of relational or social ethics in action: plain-language consent fosters informed participation; disclosure of monetisation makes visible the economic flows underpinning evidence generation; governance with patient representation redistributes epistemic authority; visible benefit-sharing acknowledges the collective origins of data value; and protocol registration transforms accountability into a traceable public record. Together, these measures link ethical theory to institutional design.

### Critical appraisal of the five safeguards: strengths, constraints, and alternatives

While the five safeguards proposed in this commentary represent pragmatic extensions of relational and social ethics, their implementation introduces important operational considerations that require careful design. Transparent consent models must balance clarity with usability, particularly when secondary data uses are numerous or evolving. Experience from UK initiatives such as care.data and NHS app frameworks demonstrates that layered consent approaches are both feasible and informative, while also highlighting the importance of managing user burden and engagement over time. Mandatory disclosure of monetisation models can encounter proprietary-data constraints, though pilot registries (e.g., Health Data Research UK's Innovation Gateway) suggest that summary-level transparency is achievable. Patient representation in governance requires training and institutional support to avoid tokenism, as seen in early NHS data board pilots. Reinvestment mechanisms face definitional and administrative hurdles regarding “fair value” of data, though international experience (e.g., Canada Health Infoway, France's Health Data Hub) illustrates models for reinvestment without direct remuneration. Finally, mandatory registration of non-interventional studies may increase administrative burden; however, parallels with clinical trial registration suggest that transparency gains outweigh compliance costs.

Alternative models—such as dynamic consent, data cooperatives, or public-private data trusts—offer complementary mechanisms to enhance accountability but remain less standardised or scalable across jurisdictions. The five safeguards proposed here were therefore prioritised for their cross-jurisdictional feasibility, institutional maturity, and immediate applicability within existing EMA/FDA/HTA frameworks.

Beyond formal governance, legitimacy also depends on narrative coherence—the stories institutions tell about why and how data are used. Public trust is sustained not only by compliance but by shared meaning. When narratives emphasise partnership, reciprocity, and societal value, they generate alignment; when they focus narrowly on efficiency or profit, they breed scepticism. Embedding narrative transparency into institutional communication—through patient-facing dashboards, co-created public reports, or community advisory panels—can transform technical openness into moral credibility (26, 27).

The EHDS now offers a historic opportunity to embed these relational and social dimensions at scale. By linking cross-border health data, EHDS can enable transformative public health insights, but without deliberate attention to fairness and reciprocity, it risks amplifying the very inequities it seeks to overcome. The success of EHDS will therefore hinge on whether its implementation includes patient representation in governance boards, clarity around data valuation, and reinvestment mechanisms that channel benefits back to citizens.

Embedding these five safeguards into RWE practice would extend the concept of evidence integrity from methodological adequacy to social legitimacy. This reframing redefines “high-quality evidence” as data that are not only accurate and reproducible but also ethically sourced, transparently governed, and socially reciprocated. In that sense, RWE's future credibility will depend as much on the fairness of its data relationships as on the rigour of its statistics. Only by uniting these two dimensions—scientific validity and moral legitimacy—can the promise of real-world data be realised in full.

Each safeguard introduces specific trade-offs: consent models may reduce scalability, disclosure requirements may conflict with proprietary constraints, governance structures require sustained investment, and benefit-sharing mechanisms raise questions regarding implementation and valuation. However, these considerations should not be interpreted as barriers but as necessary design elements in building trustworthy and sustainable data ecosystems. The convergence of findings across patient surveys, policy analyses, and governance case studies consistently highlights that trust is not secured through compliance alone, but through visible fairness, transparency, and reciprocity in how data are used and governed. In this context, embedding these safeguards within existing RWE frameworks represents a pragmatic and achievable extension of current practice rather than a fundamental restructuring. A structured comparison of current RWE and HTA frameworks against key ethical dimensions is presented in Table 1.

## Conclusion

RWE cannot thrive on methodological excellence alone. Patients' willingness to share—and society's mandate to use—rests on trust that secondary uses (including monetised ones) are transparent, fairly governed, and deliver visible public benefit. With EHDS now in force and global transparency tools maturing, the moment is ripe to embed reciprocity into the RWE ecosystem.

The five safeguards outlined in this commentary—plain-language consent, disclosure of monetisation models, governance with patient representation, visible benefit-sharing, and mandatory protocol registration—provide a feasible framework to achieve this.

This commentary focuses on advancing ethical legitimacy as a complementary dimension to methodological and operational excellence in real-world evidence ecosystems.

Fair governance of real-world data is therefore not merely a regulatory aspiration but a moral contract grounded in *relational ethics* and the *social responsibility of science*. As data ecosystems grow more interconnected, the legitimacy of RWE will depend as much on relational integrity as on analytical precision. Building mechanisms for dialogue, transparency, and visible benefit-sharing can convert data contribution from an act of compliance into one of trust and shared purpose.

The future of RWE will ultimately be measured not only by the robustness of its methods, but by the fairness of its relationships—with patients, with publics, and with the systems that rely on their information. As EHDS and comparable frameworks are operationalised, embedding these safeguards offers a practical route to ensuring that large-scale secondary data use retains its social licence. Perhaps it is time to move beyond passive consent and towards a new call for accountability—one that could be captured in a simple but powerful reminder: “That is MY DATA”.

Future research should empirically test the feasibility and acceptability of these safeguards through pilot RWE programmes under the EHDS and comparable national frameworks, ensuring that ethical principles evolve alongside scientific progress.
